# Memoir of Dr. Hope

**Published:** 1841-07

**Authors:** 


A Sketch of the Character of James Hope, M.D., F.R.S., late one of the Physicians to St. George's Hospital, with brief preliminary notices of his Life.

James Hope, the ninth child of Thomas Hope, Esq., of Prestbury Hall, Cheshire, was born at Stockport on the 23d of February, 1801. Very early indications of quickness and intelligence, of general activity, and of a mild, gentle disposition, gave a pleasing promise of future excellence.

Under an inefficient instructor, he was left, until nearly eleven years of age, to pursue in a great degree his own inclinations in reading. His avidity for knowledge, however, led him to turn over his father's books with intense interest, and to select for his perusal not only the productions of fancy and amusement, but also works of solid instruction and even of high genius. When about eight years old, Milton's Paradise Lost engaged his attention, as also Parkes' Chemical Catechism, on which he happened to stumble. He became so engrossed with chemistry as at once to commence a zealous experimentalist, and was not a little mortified, after many trials, to find himself unable to make gunpowder for the supply of his sporting brothers.

From the age of eleven to seventeen and a half, he made steady advances in his classical studies under able masters; and having determined, if possible, to be a first-class man at college, he did not relax his vigorous application until he had mastered the highest Greek authors. During this period he was also storing his mind with general literature, and becoming well acquainted with works of taste.

Having a great desire to practise at the English bar, when the period for his leaving school arrived he was much disappointed to find that his father had neglected to secure him a place at Oxford, and was bent on his entering on mercantile pursuits. This Dr. Hope positively refused to do, and after a year's delay a compromise was made. His father offered him the medical profession, and though he had a strong repugnance to it he consented on condition that he should be permitted to practise in London. Whilst waiting for rooms, he resided at Oxford for a year and a half. His father, tired of this delay, insisted upon his commencing his medical education, and being thwarted in his cherished wish of completing his studies at Oxford, he proceeded to Edinburgh. At that celebrated school of medicine he pursued a laborious and highly successful course of study during five years; graduating with much eclat, and during the latter portion of the time holding, with signal practical advantage to himself and others, the offices of house-physician and house-surgeon at the Royal Infirmary.

From Edinburgh he went to London, with a view more especially to perfect his acquaintance with practical surgery. After diligently spending six months at St. Bartholomew's Hospital, he passed the College of Surgeons, and then commenced the tour of Europe. Attracted by some most favorable opportunities of cultivating auscultation and pathological science in Paris, he tarried in that capital an entire year. Thence he visited the principal cities and other interesting localities of Switzerland and Italy, and returned to England after an absence of two years. In December, 1828, Dr. Hope fixed himself as a physician in Lower Seymour-street, Portman-square.

In 1831 he married Anne, daughter of J. W. Fulton, Esq., of Upper Harley-street, formerly of Calcutta. One child (a son) resulting from that happy union survives his lamented father. The same eventful year was marked by his election as senior physician to the Marylebone Infirmary, which office he held three years, only relinquishing it on his appointment as assistant physician at St. George's Hospital. That laborious field of exertion proved overwhelming. In the course of five years he saw 20,000, and took notes of 15,000 out-patients which he had there attended.

In 1839, on the resignation of Dr. Chambers, he was chosen physician to that hospital; but his exhausting duties in his former capacity had broken down the powers of his constitution. Phthisis supervened. At the age of forty, and on the 13th of May last, this amiable and accomplished physician departed from our world. In 1832 Dr. Hope was made a fellow of the Royal Society. In the succeeding years he received diplomas from several foreign societies, and in 1840 the College of Physicians elected him a fellow of their body.

By his will he directed that a post-mortem examination should be made, and that the persons employed should be at liberty to make any use of the morbid appearances they might think fit for the benefit of science, assigning as his reason that a physician should set a good example. The examination was accordingly made under the direction of Drs. Latham and Watson, who during his long illness had attended him with an unremitted attention, a sympathy, and a kindness, honorable alike to themselves and to their profession.

We proceed to give an outline of Dr. Hope's character, as viewed in its intellectual, moral, and professional aspects.

Dr. Hope's intellectual qualities were of a high order. A quick perception, a ready and singularly retentive memory, clearness, vigour, and comprehensiveness of thought, were happily associated with a sound and discriminating judgment, great decision, and a modest independence of mind.

From an early period Dr. Hope evinced a power of giving close and continuous attention to any subject in which he was engaged. Hence the impressions made upon his mind were singularly clear, and his recollections proportionally vivid and accurate.

*Decision of mind* was strikingly characteristic, especially as viewed in connexion with that modest opinion of himself which accompanied him through life.

The *vigorous activity* of Dr. Hope's mind rendered occupation always agreeable. He loved information for its own sake, and scarcely less for the pleasure he found in its pursuit; and having proposed to himself a high standard of attainment, he was never satisfied with any degree of excellence short of that which he believed himself able to reach.

*Comprehensiveness* was equally an attribute of Dr. Hope's mind. With an aptitude for observing the details of science he combined an admirable facility in classifying his facts, estimating their absolute and relative value, and cautiously deducing from them general principles.

He was also distinguished by *logical acumen.* Being himself a fair and close reasoner, never descending to sophistry or equivocation, he readily detected any fallacy in argument; while his thorough acquaintance with the laws of evidence led him to require the most complete proof of which the subject in question was susceptible.

*Versatility* moreover was happily associated in Dr. Hope's mental constitution, with his power of abstraction. Such was the elasticity of his mind that he could immediately rise, as with a bound, from the gravest to the most cheerful subject. During an extended classical education, and while studying the highest Greek authors, he was equally distinguished on the play-ground as the champion of athletic games; and subsequently, when zealously devoting himself to professional studies, his natural talent for drawing was so happily cultivated in the short intervals of relaxation as to enable him to copy, amongst others, a small landscape of Vanderveld with a spirit and fidelity worthy of an accomplished artist. This production of his pencil has a place in the valuable collection of the Lord President Hope.

Having thus cursorily noticed the particular features of Dr. Hope's intellectual character, it is but right to advert to the *singularly happy proportion* which subsisted between its several faculties, of which no one seemed to overpower or eclipse another. On the contrary, each appeared to sustain and invigorate the rest; and the harmonious working of the mind remarkably corresponded with the symmetrical arrangement of all its parts. Unity of design and consistency of action pervaded Dr. Hope's proceedings. His plans were wisely conceived, and as judiciously matured. At the fitting season they were carried into effect with determined perseverance, however obstructed by difficulties or delayed by disappointments. Nor could he be induced to swerve from his fixed purpose by the lure of pecuniary advantage or the prospect of an ephemeral fame.

In its *moral* aspect Dr. Hope's character appears to great advantage.

The mild benignity of his countenance, blended as it was with an expression of acute intelligence, gave a true indication of the liveliness of his feelings and the kindness of his heart. His benevolence indeed would have frequently led him into extremes had he not, in the exercise of a wise self-discipline, accustomed his feelings to the due control of his judgment.

That Dr. Hope was ambitious cannot be denied; but his ambition was of the highest kind. He was ambitious of excellence, and was far more anxious *to be* good and great than to be thought so; and such was the noble elevation of his character that if undeserved distinctions had been within his reach he would have rejected them with disdain. To rise by legitimate means was doubtless his desire, but to rise in appearance only, or by the depression of others, was as foreign to the generosity of his nature as to his established principles of rectitude and honour.

Simplicity, ingenuousness, and magnanimity were strikingly manifested in his spirit and demeanour.

In the early stages of his career he was accustomed to view human nature in too favorable a light, an error not unfrequently attaching to young persons of high moral worth; and even when, by painful experience, he gained a deeper insight into the selfishness, double-mindedness, and envy which, alas! too often lurk even under a fair and polished exterior, he still endeavoured to put the kindest construction which truth would admit on human motives and actions. The tortuous policy of other men produced no change in him; his integrity sustained him. The directness and stability of his principles never allowed him to turn aside from the plain path of honour, even to secure the most important or favorite object. He knew himself too well to imagine that any advantage unworthily obtained could yield him true or lasting satisfaction.

Yet such was the kindness of his spirit, and so exquisitely keen his sense of fairness and justice, that the ungenerous treatment which he sometimes received even from those from whom he had deserved better things, pierced him to the quick. Yet under such trying circumstances his magnanimity ever rose superior to all other considerations, and forbade him to retaliate on the offending party.

Dr. Hope was moreover strictly conscientious. An inflexible regard to truth and justice pervaded his whole conduct. He considered himself as constantly under the eye of Omniscience, and as responsible for the right improvement of his time, talents, property, and influence. In short, he was a Christian, in the highest sense of the term, not from education merely, but after full and deliberate investigation. The momentous subject of religion demanded, he was aware, his deepest attention. Accordingly, he brought to its examination the same concentration of effort, the same patient scrutiny, the same logical acuteness, the same determination to believe nothing without conclusive evidence, which had proved availing in all his scientific researches. The result was a firm and abiding conviction of the Divine origin of Christianity, as revealed in the sacred records. Thus convinced, he unhesitatingly bowed to the authority of inspiration, and implicitly received its holy doctrines as the foundation of his highest hopes, and its holy precepts as the invariable rule of his conduct. Nor is it superfluous to add, that his confidence in the religion of the Gospel remained unshaken to the last. It proved his highest solace in affliction, and his support in death.

The *professional* character of Dr. Hope strikingly exemplified the practical influence of the valuable qualities already specified.

The profession of physic was not in the first instance his own choice. On the contrary, he had entertained a strong repugnance to it. But having at length resolved to be a physician, he immediately brought all the energies of his mind to bear upon the one point, of thoroughly qualifying himself for an elevated position in the science and practice of medicine. His decision of character led him at once to impose proper limits on his favorite studies in general literature and the fine arts; in which, too, he had made considerable proficiency. He soon, however, had occasion to apply his skill as a draughtsman and colourist to the illustration of interesting subjects in pathology; and thus, by the skill and fidelity of his pencil, as well as by his close observation of disease, he not only contributed largely to the advancement of science, but also laid a deep and broad foundation for future eminence and usefulness. All the drawings in his plates of morbid anatomy were executed by his own hand.

Nature also seemed to have peculiarly qualified Dr. Hope for auscultation. His hearing, as well as his vision, was remarkably acute. With an exquisite ear for music he could distinguish the slightest differences in sound, and could distinctly perceive sounds which were so faint as to be inaudible to most other persons. This important faculty, aided by a long-continued habit of closely observing the *general* indications of disease, eventually raised him to the highest distinction as an auscultator.

With such endowments and such principles of action, it will be readily supposed that Dr. Hope's career of academical studies must have given a high promise of professional excellence; and such was the fact. The same studious habits were uniformly continued during the whole period of actual practice. He was constantly accumulating facts and educing principles. On settling in London, having only *one* private acquaintance, his professional efforts for a few years were attended with scarcely any pecuniary advantage; yet was he never disheartened, but persevered with cheerful alacrity and untiring diligence in adding to his stores of practical information; investigating doubtful points; submitting theories to the test of rigid induction; and more especially, proceeding steadily with his two great works on the Diseases of the Heart, and on Morbid Anatomy. When the first of these was published, about nine years since, the whole profession united in commendation of its excellence; and, in the enlarged and improved form in which the author was fortunately enabled to reproduce it, in a third edition, two years before his death, it is now universally acknowledged to be the best book on the subject in any language. Dr. Hope became at once a high authority in that class of maladies. His brethren and the public flocked to him; and had he not prematurely broken down under the pressure of excessive labour, an ample fortune and the highest professional elevation, would in all probability have been his reward.

Dr. Hope's demeanor in the sick chamber was peculiarly appropriate. A kind, attentive interest, combined with a calm, well-sustained investigation of the case, secured his patient's confidence and regard.

As a lecturer, Dr. Hope was highly prized. A familiar acquaintance with his subject, and an evident desire to impart to his pupils, in the clearest and most impressive manner, the extensive information he possessed, were advantageously associated with a grateful fluency of diction, and considerable power of lively illustration. A high-toned morality, and an almost affectionate interest in the welfare of his class, inspired and obtained their high respect and attachment.

It is most gratifying to record that the celebrity thus acquired was borne with perfect equanimity. Dr. Hope continued the same modest, ingenuous, benevolent character as he had ever been. No assumption of consequence, no harshness of manner, no unkindness to junior practitioners or to the sick, whether rich or poor, ever derogated from his well-earned fame.

Dr. Hope's career was indeed a short one. At the age of forty, in the very zenith of prosperity, in the midst of honours and usefulness, with a rapidly increasing fortune, and in the full enjoyment of domestic happiness, he was called to resign them all. But futurity was not a blank to him; he had a hope full of immortality.

Our limits have necessarily excluded almost every kind of proof and illustration. Both, however, may be expected from the rich and ample materials which are already in a course of preparation for a more extended memoir.

